# Feasibility of a CT-based lymph node radiomics nomogram in detecting lymph node metastasis in PDAC patients

**DOI:** 10.3389/fonc.2022.992906

**Published:** 2022-10-05

**Authors:** Qian Li, Zuhua Song, Dan Zhang, Xiaojiao Li, Qian Liu, Jiayi Yu, Zongwen Li, Jiayan Zhang, Xiaofang Ren, Youjia Wen, Zhuoyue Tang

**Affiliations:** ^1^ Department of Radiology, Chongqing Medical University, Chongqing, China; ^2^ Chongqing Institute of Green and Intelligent Technology, Chinese Academy of Sciences, Chongqing, China; ^3^ Chongqing School, University of Chinese Academy of Sciences, Chongqing, China; ^4^ Department of Radiology, Chongqing General Hospital, Chongqing, China

**Keywords:** pancreatic ductal adenocarcinoma, lymph node metastasis, radiomics, nomogram, computed tomography

## Abstract

**Objectives:**

To investigate the potential value of a contrast enhanced computed tomography (CECT)-based radiological-radiomics nomogram combining a lymph node (LN) radiomics signature and LNs’ radiological features for preoperative detection of LN metastasis in patients with pancreatic ductal adenocarcinoma (PDAC).

**Materials and methods:**

In this retrospective study, 196 LNs in 61 PDAC patients were enrolled and divided into the training (137 LNs) and validation (59 LNs) cohorts. Radiomic features were extracted from portal venous phase images of LNs. The least absolute shrinkage and selection operator (LASSO) regression algorithm with 10-fold cross-validation was used to select optimal features to determine the radiomics score (Rad-score). The radiological-radiomics nomogram was developed by using significant predictors of LN metastasis by multivariate logistic regression (LR) analysis in the training cohort and validated in the validation cohort independently. Its diagnostic performance was assessed by receiver operating characteristic curve (ROC), decision curve (DCA) and calibration curve analyses.

**Results:**

The radiological model, including LN size, and margin and enhancement pattern (three significant predictors), exhibited areas under the curves (AUCs) of 0.831 and 0.756 in the training and validation cohorts, respectively. Nine radiomic features were used to construct a radiomics model, which showed AUCs of 0.879 and 0.804 in the training and validation cohorts, respectively. The radiological-radiomics nomogram, which incorporated the LN Rad-score and the three LNs’ radiological features, performed better than the Rad-score and radiological models individually, with AUCs of 0.937 and 0.851 in the training and validation cohorts, respectively. Calibration curve analysis and DCA revealed that the radiological-radiomics nomogram showed satisfactory consistency and the highest net benefit for preoperative diagnosis of LN metastasis.

**Conclusions:**

The CT-based LN radiological-radiomics nomogram may serve as a valid and convenient computer-aided tool for personalized risk assessment of LN metastasis and help clinicians make appropriate clinical decisions for PADC patients.

## Introduction

Pancreatic ductal adenocarcinoma (PDAC), an aggressive malignancy, is expected to become the second leading cause of cancer deaths worldwide by 2030, of which 5-year overall survival rate is still as low as 9% (1). Despite advances in therapeutic methods, radical resection with appropriate lymphadenectomy remains the only curative method. Lymph node (LN) metastasis, one of the strongest postoperative prognostic indicators, is closely associated with poor prognosis (2–5). In clinical practice, the extent of lymph node dissection in pancreatic cancer remains controversial, including extended and standard lymphadenectomies. Preoperative diagnosis of LN metastasis plays a crucial role in selecting a reasonable LN dissection method, which could not only avoid the omission of metastatic LNs but also decrease postoperative complications and prevent overtreatment. In addition, the National Comprehensive Cancer Network guidelines recommend preoperative neoadjuvant treatment in PDAC patients with LN metastasis, which is associated with a survival benefit (6–8). Therefore, accurate preoperative diagnosis of LN metastasis plays an important role in providing individualized treatment plans for PDAC patients.

Computed tomography (CT) is the primary examination method for PDAC tumor staging in some clinical practice guidelines (9, 10). Many studies (11, 12) proposed the short-axis diameter of LN above 10 mm as a criterion to diagnose metastatic LNs; however, its diagnostic accuracy is easily influenced by enlarged LNs secondary to inflammatory hyperplasia. Other CT image features (11, 13, 14), including LN shape, border contour and heterogeneity, are utilized to improve diagnostic performance for LN metastasis in PDAC. It is worth noting that detecting these features relies on subjective judgment and may be challenging for first-line radiologists with no substantial diagnosis experience. Both qualitative and semiquantitative analyses by visual evaluation on conventional radiological features cannot accurately detect metastatic LNs, so more studies are needed for exploring preoperative diagnostic tools to detect LN metastasis in PDAC patients.

In recent years, computer-aided imaging analysis could be applied in clinic because of the sustained and fast growth of computer science. Radiomics is an emerging discipline that can rapidly extract innumerable features from medical images such as CT, magnetic resonance and ultrasound images in an automated, high-throughput manner. These features could reflect tumor heterogeneity quantitatively and the underlying pathophysiology, which are imperceptible to naked eyes (15–17). This method has been used to evaluate the LN status preoperatively in head and neck, colon, papillary thyroid, cervical and prostate cancers, with ideal predictive accuracy (18–22). In PDAC, current studies are mainly based on original tumor radiomics to predict LN metastasis (23–25), and studies based on LN radiomics to discriminate metastatic from non-metastatic LNs are scarce. In this study, we hypothesized that LN radiomic features may contribute to evaluating the LN status preoperatively and attempted to develop a contrast-enhanced CT (CECT)-based LN radiological-radiomics nomogram for detecting LN metastasis in patients with PDAC.

## Materials and methods

### Patients and LNs

This retrospective study was approved by the ethics committee of Chongqing general Hospital, and the requirement for written informed consent was waived. From January 2019 to October 2021, PDAC patients administered surgical resection with lymph node dissection in Chongqing General Hospital were retrospectively reviewed.

Inclusion criteria were (1): pathologically confirmed PDAC and LN status; (2) thin-layer CECT examination within 2 weeks before surgery. Exclusion criteria were: (1) a history of systemic treatment before surgery; (2) other coexisting primary malignancies; (3) missed clinical appointment; (4) image quality unsatisfactory for analysis.

**Figure 1:**
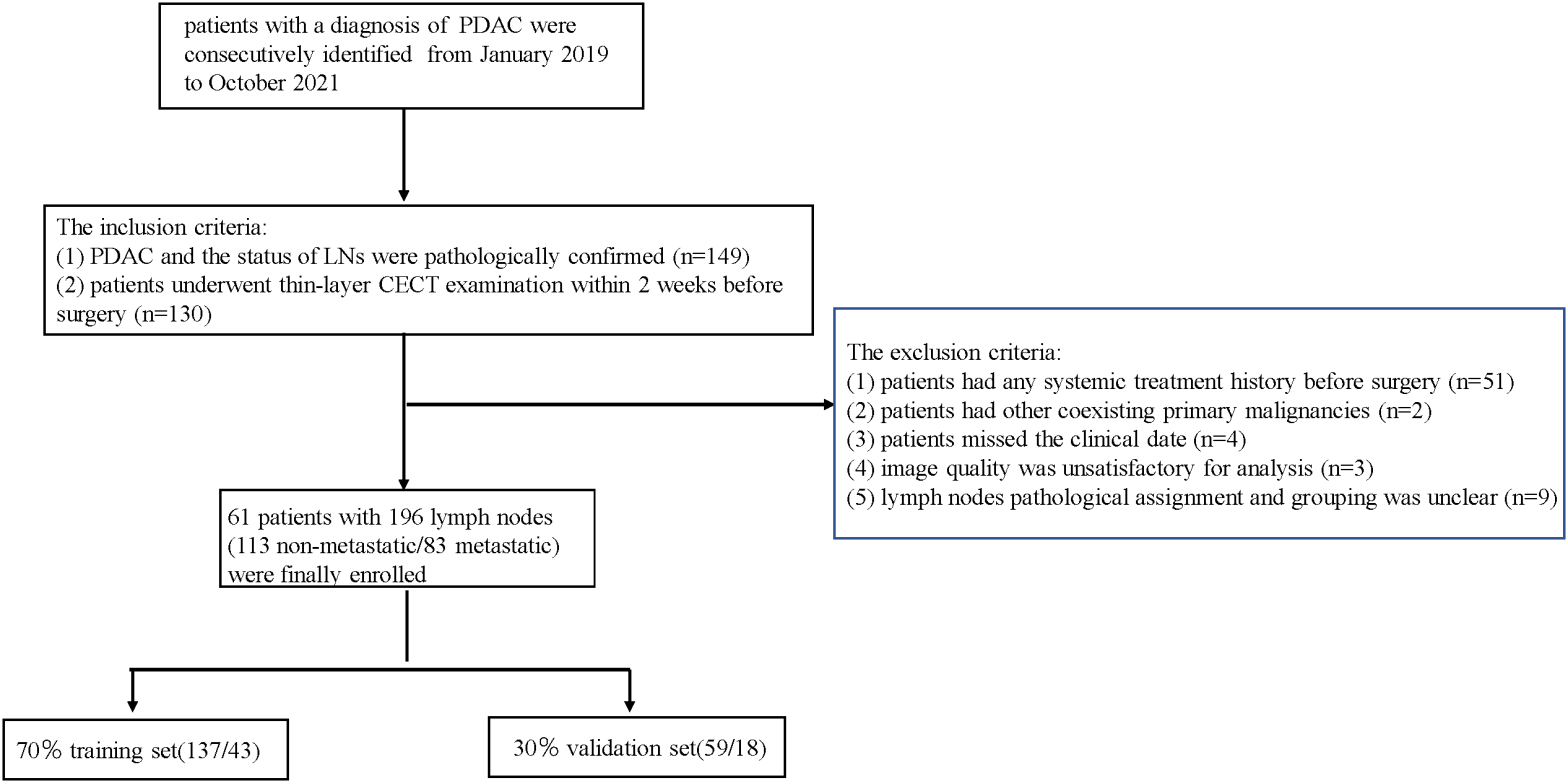
Flowchart of patients’ selection.

The Japan Pancreas Society’s nodal classification of regional lymph node stations of the pancreas was used throughout the study to describe radiological and pathological LN groups (26). The criteria for LN eligibility were: (1) when LNs in one group were all pathologically confirmed to be metastatic or non-metastatic, all the LNs of this group were included; (2) when LNs in one group contained both metastatic and non-metastatic LNs, all the LNs of this group were excluded; (3) LNs with a short-axis diameter below 5 mm were excluded. The flowchart for selecting the study population is shown in **Figure 1**.

A total of 196 LNs (113 non-metastatic and 83 metastatic) with histological confirmation in 61 patients (31 males and 30 females; mean age, 62.3 ± 9.2 years; age range, 39–80 years) were analyzed. The subjects were divided into the training (January 2020 to October 2021) and validation (January 2019 to December 2019) cohorts at a ratio of 7:3 according to the time of CT. The training cohort included 43 patients with 137 LNs, and validation cohort included 18 patients with 59 LNs.

### The CECT protocol

The CT images of 39 participants were acquired on a spectral CT scanner (IQon Spectral CT, Philips Healthcare). Typical imaging parameters were: tube voltage, 120 kV; smart mAs; rotation time, 0.5 s; detector collimation, 64 × 0.625 mm; field of view, 350 × 350 mm; matrix, 512 × 512; layer thickness, 5 mm; reconstruction thickness, 1.25 mm. A nonionic contrast medium (Iohexol, 350 mgI/ml, Schering, Berlin, Germany) was injected with an automatic injector at a dose of 1.5 ml/kg at 3.5 ml/s, followed by 30 ml of saline flashing at the same rate. Arterial phase scans were started with a delay of 10s after passing the predetermined threshold of 150 HU within the abdominal aorta (activated bolus tracking). Portal vein phase scans were started 20 s after the arterial phase.

The CT images of 22 participants were acquired on a 64-slice CT scanner (Aquilion CX, Canon Medical Systems). The same acquisition protocol was used: tube voltage, 120 kV; smart mAs; rotation time, 0.5 s; detector collimation, 64 × 0.5 mm; field of view, 350 × 350 mm; matrix 512 × 512; layer thickness, 5 mm; reconstruction thickness, 1.25 mm. The nonionic contrast medium Iohexol (350 mgI/ml) was injected with an automatic injector at a dose of 1.5 ml/kg at 3.5 ml/s, followed by 30 ml of saline flashing at the same rate. Arterial phase images were obtained 28 s after contrast medium injection, while portal venous phase scans were obtained 22 s after arterial phase image acquisition.

All images were uploaded to the picture archiving and communication system (PACS) for further examination.

### Clinical and radiological characteristics

Preoperative demographic characteristics, laboratory findings and CECT conventional features were obtained. The radiological features of LNs, including size, shape, margin, and degree and pattern of enhancement, were analyzed by two radiologists with 7 and 9 years of experience in abdominal imaging, respectively. They were blinded to pathological data and research design. Inter-reader agreement was investigated for evaluating the radiological features by intraclass correlation coefficient (ICC). The sizes of LNs were reflected by their maximal short-axis diameters. LN shapes were categorized as regular (oval) and irregular (round, spiculated or lobulated), and LN margin was blurred or clearly delineated. The enhancement patterns of LNs were categorized as homogenous or heterogeneous in the portal venous phase. The degree of enhancement was estimated with reference to the soft tissue (14, 27, 28).

### Image processing, VOI delineation, and radiomic analysis

**Figure 2:**
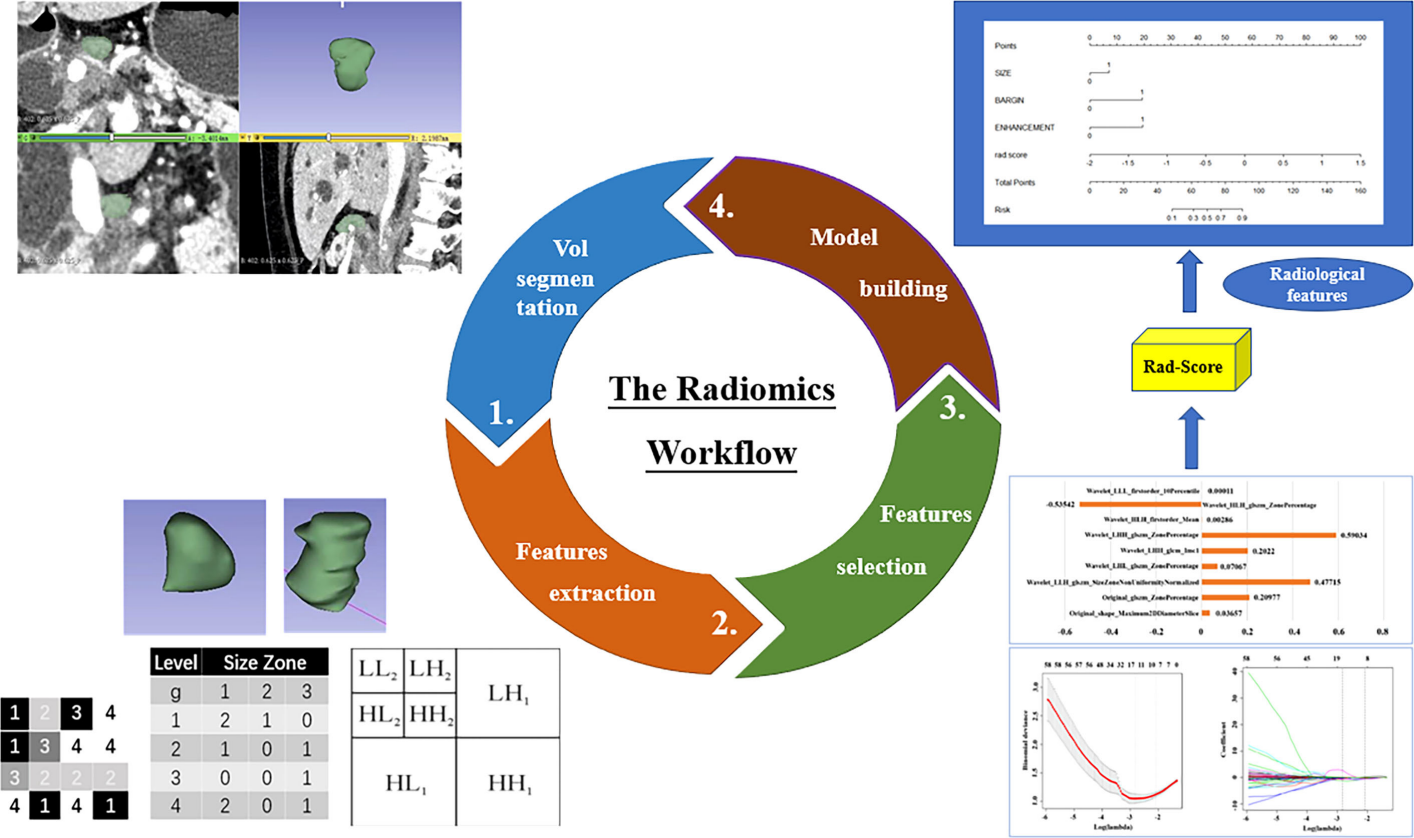
The workflow of the radiomics analysis of LNs in this study. Step 1: LNs were semi-automatically segmented slice by slice in portal venous phase images. Step 2: Radiomics features were extracted from the identified VOIs. Step 3: The LASSO logistic regression with penalty parameter tuning conducted by 10-fold cross-validation was used to select the optimal radiomics features. Step 4: The LN Rad-score and the nomogram incorporating radiological features with Rad-score were established.

This study workflow is shown in **Figure 2**. The detailed steps for Volume of Interest (VOI) segmentation and feature extraction were as follows. ① The original portal venous phase images were downloaded from the PACS (Carestream) and saved as Digital Imaging and Communication in Medicine (DICOM) files. ② The concrete steps for image normalization were: (a) for image registration, every portal venous phase image slice from the raw data was resampled to a standardized pixel dimension size of 1.0 × 1.0 × 1.0 mm^3^; (b) for gray-level discretization, the image intensity of every portal venous phase image was normalized *via* the gray-level discretization method with a fixed number of bins (256 bins); (c) portal venous phase images were viewed in a fixed head window (level = 60 Hounsfield unit (HU); width = 400 HU). ③ VOIs were acquired by sketching LN borders manually slice-by-slice on axial sections, automatically merged into a 3D region using an open source software (3D Slicer, version 4.13.0; Boston, MA, USA), and adjusted on sagittal and coronal sections by a radiologist with 7 years of abdominal diagnosis experience. ④ All VOIs were confirmed again by a senior radiologist with 20 years of abdominal diagnosis experience. ⑤ The radiomic features were automatically extracted from the VOIs with SlicerRadiomics (an extension for 3D-slicer).

Finally, a total amount of 1037 radiomic features in eight categories (first-order, shape-based histogram, gray-level cooccurrence matrix (GLCM), grey level size zone matrix (GLSZM), gray level run length matrix, gray level dependence matrix, neighboring grey-tone difference matrix and wavelet-based features) were extracted from each VOI automatically. A two-step program was designed to reduce high-dimensional data and avoid overfitting. First, features with ICC above 0.8 were considered to be reproducible and stable. Secondly, the least absolute shrinkage and selection operator (LASSO) algorithm was used to select optimal features with nonzero coefficients, with parameter tuning performed with 10-fold cross-validation to screen optimal radiomic features.

### Model building

The differences in CT radiological features between metastatic and non-metastatic LNs were first compared by univariate analysis. Subsequently, significant (P<0.05) radiological features in the univariate analysis were entered into multivariate analysis to determine independent predictors of metastatic LNs and to develop a radiological model by multivariate logistic regression (LR) analysis in the training cohort. Odds ratio (OR) and 95% confidence interval (CI) for each independent factor were calculated.

A radiomics score (Rad-score) was calculated for each LN by determining the linear combination of the optimal radiomic features weighted by their respective LASSO coefficients in the training cohort. The Rad-score model for assessing the metastatic LNs of PDAC patients was first developed in the training cohort.

To provide a simple tool for clinicians to predict metastatic LNs in PDAC, a radiological-radiomics nomogram, which combined the LN Rad-score and independent LN’s radiological features, was built by multivariate LR analysis in the training cohort.

### Model evaluation

The diagnostic performances of the three models were assessed in the validation cohort by receiver operating characteristic (ROC) curve analysis. The area under the ROC curve (AUC), sensitivity and specificity were all determined, and the Delong test was performed to compare the AUCs of the three models. The calibration ability of the radiological-radiomics nomogram was assessed by calibration curve analysis in the whole cohort, which could compare consistency between the pathological findings of LNs and nomogram-evaluated outcomes. The clinical values of the Rad-score model and the radiological-radiomics nomogram were assessed using decision curve analysis (DCA) by calculating the net benefits in the training and validation cohorts for a range of threshold probabilities (29).

### Statistical analysis

Statistical analysis was performed with the R software (http://www.R-project.org), MedCalc (version 18.2.1), SPSS (version 25.0) and empower (R) (www.empowerstats.com, X&Y Solutions, Inc., Boston, MA). Baseline clinical characteristics were expressed as mean ± standard deviation, or number and percentage, as appropriate. The two-sample t test was performed to compare continuous variables. The chi-squared test was carried out to compare categorical variables. A two-sided P value below 0.05 was considered statistically significant. Inter-reader agreement was calculated using ICC analysis.

## Results

### Patient characteristics and LNs’ radiological features

The clinical characteristics of the training and validation cohorts are summarized in **Table 1**. There were no significant differences between the training and validation cohorts in clinical characteristics. The radiological features of metastatic and non-metastatic LNs in the training cohort are summarized in **Table 2**. Agreement for evaluating radiological features between two radiologists was good to excellent overall. ICC of LN margin, size, shape, density and pattern of enhancement were 0.871, 0.906, 0.892, 0.963 and 0.906, respectively. After univariate and multivariate analyses, LN size(odds ratio [OR], 5.025; 95% CI, 1.453 - 17.374; P = 0.011), LN margin(OR, 7.482; 95% CI, 2.705 - 20.696; P < 0.001)and enhancement pattern (OR, 7.039; 95% CI, 2.107 - 23.513; P = 0.002) showed statistically significant differences, and were included as independent predictors of LN metastasis to construct a radiological model. The diagnostic performance of the radiological model was moderate, with an AUC of 0.831(95% CI, 0.761 - 0.900), a sensitivity of 0.632 and a specificity of 0.900 in the training cohort, and an AUC of 0.756(95% CI, 0.629 - 0.884), a sensitivity of 0.615 and a specificity of 0.848 in the validation cohort (**Figure 4**).

**Table 1:** Patients characteristics.

Characteristic	Training Cohort (n = 43)	Validation Cohort (n = 18)	p
Gender, No. (%)			0.943
male	22 (51.2)	9 (50)	
female	21 (48.8)	9 (50)	
Age (Mean ± SD)	62.56 ± 8.797	62.11 ± 10.51	0.865
CA199 level, No. (%)			0.276
Abnormal	33 (76.7)	16 (88.9)	
Normal	10 (23.3)	2 (11.1)	
CA125 level, No. (%)			0.147
Abnormal	32 (74.4)	10 (55.6)	
Normal	11 (25.6)	8 (44.4)	
Location, No. (%)			0.924
Head and neck	12 (27.9)	5 (27.8)	
body	21 (48.8)	8 (44.4)	
tail	10 (23.3)	5 (27.8)	

**Table 2:** Univariate and multivariable analysis of CT radiological features for LN metastasis evaluation in the training cohort.

Factors	Univariate analysis			Multivariate analysis		
	Odds ratio	(95% CI)	*p*	Odds ratio	(95% CI)	*p*
Size	11.943	3.827-37.272	< 0.001	5.025	1.453-17.374	0.011
Shape	1.700	0.773-3.741	0.187			
Margin	10.000	4.080-24.512	< 0.001	7.482	2.705-20.696	< 0.001
Degree of enhancement						
Mild	ref.					
Moderate	2.273	0.930-5.554	0.072			
Strong	1.773	0.718-4.377	0.214			
Patterns of enhancement	11.401	3.768-34.500	< 0.001	7.039	2.107-23.513	0.002

### Rad-score and radiological-radiomics nomogram construction and performance evaluation

**Figure 3:**
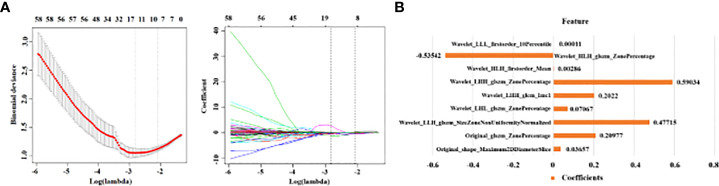
The framework for radiomics features selection. **(A)** The LASSO logistic regression was used to select LN radiomics. A tuning parameter was selected *via* 10-fold cross-validation and nine with nonzero coefficients were selected finally. **(B)** Histogram shows the role of nine selected radiomics features used to calculate the Rad-score. The y-axis represents individual radiomics features, with their coefficients in the LASSO regression analysis plotted on the x-axis.

**Figure 4:**
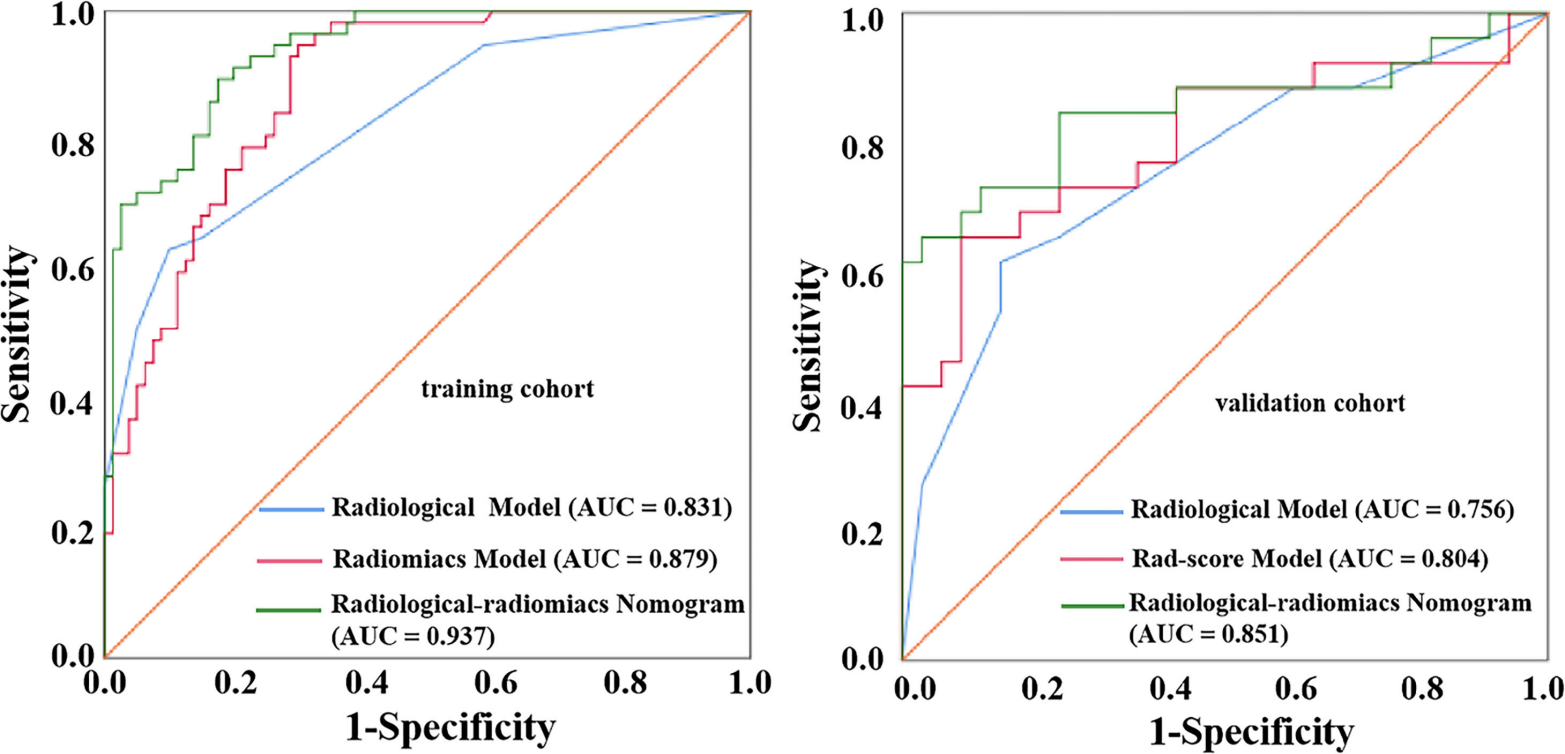
ROC curves of the radiological model, Rad-score model and radiological-radiomics nomogram for diagnosing metastatic LNs in the training cohort and validation cohort.

To identify PDAC patients with LN metastasis, a total of 9 most predictive radiomic features with nonzero coefficients in the LASSO algorithm were finally selected (**Figure 3**) and incorporated into the Rad-score model. The formula was as follows: Rad-score = 0.2098 × Original_glszm_ZonePercentage + 0.0366 × Original_shape_Maximum2DDiameterSlice + 0.0001Wavelet_LLL_firstorder_10Percentile + 0.029Wavelet_HLH_firstorder_Mean + 0.2022 × Wavelet_LHH_glcm_Imc 1 + (− 0.5354) × Wavelet_HLH_glszm_ZonePercentage + 0.0707 × Wavelet_LHL_glszm_ZonePercentage + 0.5903 × Wavelet_LHH_glszm_ZonePercentage + 0.4772 × Wavelet_LLH_glszm_SizeZoneNonUniformityNormalized. The Rad-score model showed a better performance than the radiological model the radiological model, with an AUC of 0.879(95%CI, 0.824 - 0.934), sensitivity of 0.684 specificity of 0.838 in the training cohort, and an AUC of 0.804(95%CI, 0. 685 - 0.924), sensitivity of 0.654, specificity of 0.818 in the validation cohort (**Figure 4**). The DeLong test displayed that there was no significant differences between the AUCs of the radiological model and Rad-score models in the training cohort (P = 0.258, DeLong test) and validation cohort (P = 0.543, DeLong test).

**Figure 5:**
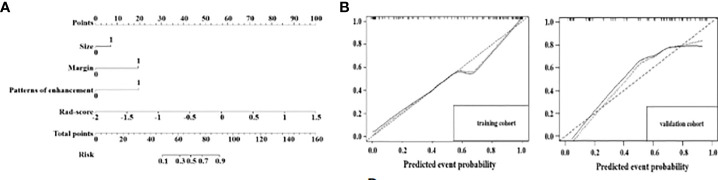
A radiological-radiomics nomogram was plotted combining independent radiological features with Rad-score in the training cohort **(A)**. Calibration curves for the radiological-radiomics nomogram in the training cohort and in the validation cohort **(B)**. The 45° straight line indicates the ideal performance of the radiological-radiomics nomogram. A closer distance between two curves indicates higher accuracy.

Three radiological features (LN size, LN margin and enhancement pattern) combined with the LN Rad-score were used to build a radiological-radiomics nomogram by multivariate LR analysis. The radiological-radiomics nomogram for identifying PDAC patients with LN metastasis risk in the training cohort is shown in **Figure 5A**. This nomogram showed an AUC of 0.937 (95%CI, 0. 900 - 0.974), a sensitivity of 0.772 and a specificity of 0.863 in the training cohort, and an AUC of 0.851(95%CI, 0.741 - 0.961), a sensitivity of 0.692 and a specificity of 0.909 in the validation cohort (**Figure 4**). The DeLong test revealed that the radiological-radiomics nomogram had enhanced predictive performance than the radiomics and radiological models in the training cohort(P = 0.010, DeLong test; P < 0.0001, DeLong test), with no significant differences in the validation cohort(P = 0.228, DeLong test; P = 0.084, DeLong test).

Calibration curve analysis of the nomogram demonstrated the prediction results were in good agreement with the actual observations both in the training and validation cohorts (**Figure 5B**).

**Figure 6:**
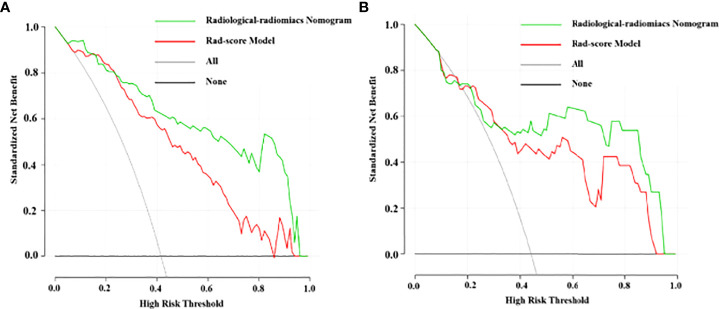
DCA for the Rad-score model and radiological-radiomics nomogram in the training cohort **(A)** and validation cohort **(B)**. The y-axis measures the net benefit and the x-axis represents the threshold probability. The grey line that all patients had LN metastasis and the black line indicate no patients had LN metastasis. The red line and the green line indicate the net benefit of the Rad-score model and the radiological-radiomics nomogram at different threshold probabilities, respectively. The radiomics nomogram had a higher overall net benefit in differentiating LN metastasis than Rad-score model.

DCA of the Rad-score model and radiological-radiomics nomogram in the training and validation cohorts are presented in **Figure 6**. The curves demonstrated that the Rad-score model and radiological-radiomics nomogram provided more benefit than the treat all or none principle in PDAC patients for all threshold probabilities in the training cohort and all most threshold probabilities in the validation cohort.

## Discussion

In this retrospective study, we developed a radiological-radiomics nomogram that incorporated the LN radiomics signature with LNs’ radiological features to evaluate the status of LNs in patients with PDAC. In addition, the radiological-radiomics nomogram performed better than the Rad-score and radiological models. These findings indicated that LN radiomics analysis could be effective for preoperative diagnosis of metastatic LNs in patients with PDAC.

In the current study, we found LN size, LN margin and enhancement pattern were the optimal radiological features to detect LN metastasis. Heterogeneous enhancement, which may reflect unevenly distributed tumor angiogenesis and internal necrosis, is considered a reliable feature of LN metastasis (27, 28). Blurred margin may be caused by tumor cell infiltration into peri-nodal adipose tissue. A short-axis diameter of LNs greater than 10 mm is widely used to diagnose nodal involvement in PDAC. In agreement, we also demonstrated that large LNs (>10 mm) are more prevalent in metastatic LNs compared with non-metastatic LNs. Roche et al. also indicated this criterion could lead to high diagnostic specificity in evaluating the LN status (30). Based on these three predictors we constructed a radiological model to identify metastatic LNs in patients with PDAC, which had a good diagnostic performance with AUCs of 0.831 and 0.756 in the training and validation cohorts. These results revealed that the radiological model could be used for differential diagnosis between metastatic and non-metastatic LNs. However, texture heterogeneity as a fundamental feature of the LN itself through naked-eye observation has not been applied (31, 32).

Radiomics can extract high-dimensional objective and quantitative features from the segmented volumes and to assess the spatial distribution of voxels that could profile heterogeneity (17). The present study applied LN radiomics to evaluate the LN status and found 9 radiomic features quantified on portal-venous CT-image that could help diagnose metastatic LNs in PDAC. Among these features, most were determined on images preprocessed with wavelet filters (33), which indicated that higher order statistics features are more valuable for evaluating the LN status. Five GLSZM features could imply the extent of the spatial correlation or uniformity of gray-levels. GLCM Informational Measure of Correlation 1 was increased in metastatic LNs, which indirectly indicated that metastatic LNs have a higher degree of heterogeneity. First order features, including mean and 10^th^ percentile, significantly correlate with the LN status, which provide statistical information on the distribution and number of pixels with the same intensity in the VOI (34). Furthermore, this study found that Maximum2D Diameter Slice, which can express the largest pairwise Euclidean distance between LN surface voxels in the slice plane, was larger in the metastasis group. This was a reasonable evidence that metastatic LNs are preferably larger.

The Rad-score model composed of the above radiomic features showed better diagnostic performance compared with the radiological model, with AUCs of 0.879 and 0.804 in the training and validation cohorts, respectively.

By incorporating the radiological model and the Rad-score, we built a LN radiological-radiomics nomogram, which had the highest AUC (training cohort, 0.937; validation cohort, 0.851) and diagnostic sensitivity (training cohort, 0.772; validation cohort, 0.692). This nomogram showed significantly better diagnostic efficacy for metastatic LNs in patients with PDAC compared with the Rad-score and radiological models in the training cohort (all P<0.05, DeLong test). This finding revealed that combining the internal texture heterogeneity and radiological features of LNs could be a prospective approach to enhance precision medicine. Furthermore, this nomogram could conveniently and visually estimate the status of LNs in PDAC patients. With the help of the radiological-radiomics nomogram, individualized risk assessment in terms of detecting LN metastasis could be implemented for PDAC patients. Finally, the calibration curve of the nomogram showed good agreement between nomogram-evaluated and pathological results in the training and validation cohorts. In addition, we performed a decision curve analysis demonstrating a clinical net benefit for the radiological-radiomics nomogram. This curve showed the nomogram could confer enhanced net benefits than treating all or no patients for all threshold probabilities in the training cohort and almost all in the validation cohort.

There were several limitations in this study. Firstly, because this was a single-center retrospective study, further refinements with multicenter studies are needed to confirm these findings. Secondly, VOIs were segmented semi-automatically in this research and errors were inevitable due to segmentation uncertainty even though good agreement in inter-observer and intra-observer reproducibility was achieved. Deep learning for automatic segmentation to increase efficiency and reproducibility still needs further exploration (35). Thirdly, tumor radiomics was not included in this study, so the diagnostic value of combining the radiomics and tumor radiomic features of LNs requires further investigation. Fourthly, we only performed LN radiomics analysis based on the portal venous phase images and didn’t compare or combine with other phase images to definite which may have the potential to improve diagnostic efficiency. This owns the significance for further exploration.

## Conclusion

This study developed and validated a radiological-radiomics nomogram that integrated the LN Rad-score and LNs’ radiological features for preoperatively evaluating the LN status in PDAC patients. This nomogram could serve as an easy-to-use, noninvasive and effective tool to assist radiologists in diagnosing metastatic LNs and to guide the clinical decision-making process for PDAC patients.

## Data availability statement

The original contributions presented in the study are included in the article/supplementary material. Further inquiries can be directed to the corresponding author.

## Ethics statement

This study was approved by the ethics committee of Chongqing general Hospital, and the requirement for informed consent was waived.

## Author contributions

TZY designed and supervised the study. Qian Li conducted the experiments and drafted the manuscript. ZD revised the manuscript. RXF, WYJ and ZJY collected the imaging data. Qian Liu, LXJ and YJY analyzed the data. SZH and LZW performed statistical analysis. All the authors listed have made substantial, direct and intellectual contributions to the work and approved the manuscript for publication.

## Funding

This study was supported by the medical research Key Program of the combination of Chongqing National health commission and Chongqing science and technology bureau, China (no 2019ZDXM010) and the medical research Program of the combination of Chongqing National health commission and Chongqing science and technology bureau, China (no 2020FYYX151).

## Conflict of interest

The authors declare that the research was conducted in the absence of any commercial or financial relationships that could be construed as a potential conflict of interest.

## Publisher’s note

All claims expressed in this article are solely those of the authors and do not necessarily represent those of their affiliated organizations, or those of the publisher, the editors and the reviewers. Any product that may be evaluated in this article, or claim that may be made by its manufacturer, is not guaranteed or endorsed by the publisher.
